# Rabies in Iraq: Trends in Human Cases 2001–2010 and Characterisation of Animal Rabies Strains from Baghdad

**DOI:** 10.1371/journal.pntd.0002075

**Published:** 2013-02-28

**Authors:** Daniel L. Horton, Mashair Z. Ismail, Eman S. Siryan, Abdul Raheem A. Wali, Husam E. Ab-dulla, Emma Wise, Katja Voller, Graeme Harkess, Denise A. Marston, Lorraine M. McElhinney, Salah F. Abbas, Anthony R. Fooks

**Affiliations:** 1 Animal Health and Veterinary Laboratories Agency, Weybridge, United Kingdom; 2 Central Veterinary Laboratory, Iraq State Company for Veterinary Services, Baghdad, Iraq; 3 Baghdad Veterinary Hospital, Iraq State Company for Veterinary Services, Baghdad, Iraq; 4 National Consortium for Zoonosis Research, Leahurst, Neston, Wirral, United Kingdom; Centers for Disease Control and Prevention, United States of America

## Abstract

Control of rabies requires a consistent supply of dependable resources, constructive cooperation between veterinary and public health authorities, and systematic surveillance. These are challenging in any circumstances, but particularly during conflict. Here we describe available human rabies surveillance data from Iraq, results of renewed sampling for rabies in animals, and the first genetic characterisation of circulating rabies strains from Iraq. Human rabies is notifiable, with reported cases increasing since 2003, and a marked increase in Baghdad between 2009 and 2010. These changes coincide with increasing numbers of reported dog bites. There is no laboratory confirmation of disease or virus characterisation and no systematic surveillance for rabies in animals. To address these issues, brain samples were collected from domestic animals in the greater Baghdad region and tested for rabies. Three of 40 brain samples were positive using the fluorescent antibody test and hemi-nested RT-PCR for rabies virus (RABV). Bayesian phylogenetic analysis using partial nucleoprotein gene sequences derived from the samples demonstrated the viruses belong to a single virus variant and share a common ancestor with viruses from neighbouring countries, 22 (95% HPD 14–32) years ago. These include countries lying to the west, north and east of Iraq, some of which also have other virus variants circulating concurrently. These results suggest possible multiple introductions of rabies into the Middle East, and regular trans-boundary movement of disease. Although 4000 years have passed since the original description of disease consistent with rabies, animals and humans are still dying of this preventable and neglected zoonosis.

## Author Summary

Control of rabies requires cooperation between government departments, consistent funding, and an understanding of the epidemiology of the disease obtained through surveillance. Here we describe human rabies surveillance data from Iraq and the results of renewed sampling for rabies in animals. In Iraq, it is obligatory by law to report cases of human rabies. These reports were collated and analysed. Reported cases have increased since 2003, with a marked increase in Baghdad 2009–2010. There is no system for detecting rabies in animals and the strains circulating in Iraq have not previously been characterized. To address this, samples were collected from domestic animals in Baghdad and tested for rabies. Three out of 40 were positive for rabies virus. Comparison of part of the viral genetic sequence with other viruses from the region demonstrated that the viruses from Iraq are more closely related to each other than those from surrounding countries, but diverged from viruses isolated in neighbouring countries approximately 22 (95% HPD 14–32) years ago. Although 4000 years have passed since the original description of disease consistent with rabies, animals and humans are still dying of this preventable and neglected zoonosis.

## Introduction

The first written record of disease consistent with rabies is in the Laws of Eshunna, a Sumerian city in ancient Mesopotamia. Largely corresponding to the region of what is now the Republic of Iraq, Mesopotamia encompassed the Euphrates and Tigris river systems and is considered by many to be the birthplace of civilisation. Some of the earliest archaeological records of the domestication of dogs also originate from the area, and dogs are thought to have had religious significance during that time [1]. Those Eshunnian laws, written almost 4000 years ago, warn of fines for owners of uncontrolled ‘mad’ dogs that bite humans [2]. The association of disease with infected dogs is consistent with the knowledge that rabies is spread through the saliva of infected animals, and that dogs are the major reservoir for infections of humans in many endemic areas [3]. It wasn't until the last century, that rabies virus, a single stranded RNA virus in the family *Rhabdoviridae*, genus *Lyssavirus*, was identified as the causative agent [4].

Baghdad was established as the centre of the Arab world during the middle ages, and endured repeated changes in rule until the region came under the control of the Ottoman Empire in the 14^th^ century. Although information on rabies incidence during Ottoman rule is scarce, the speed at which the medical authorities adopted Pasteur's vaccine after the development was first published in 1885, illustrates the importance of the disease at that time [5].

The current borders of Iraq were demarcated by the Treaty of Sèvres in 1920, when the Ottoman Empire was fragmented after World War I. After a brief period under control of Great Britain, Iraq first became an independent Kingdom in 1932, and then a Republic when the monarchy was overthrown in 1958. An effective hospital based health system was developed, but was reported to have deteriorated toward the end of the 20^th^ century due to conflict, embargoes and sanctions [6], [7]. Increase in conflict from 2003 has had further effects on disease control, with migration of health professionals and restrictions on travel and resources [7].

Rabies is considered endemic in most countries in the Middle East, but establishing the true burden is prevented by a relative lack of systematic surveillance and reporting [8]. Previous studies have provided valuable insight into the molecular epidemiology of canine rabies in the Middle East, but isolates from Iraq have not previously been available for analysis [9], [10]. The Middle East and Eastern Europe Rabies Expert Bureau (MEEREB) network was established in 2010 to improve regional collaboration in rabies control and has improved exchange of information [11]. Although Iraq is not currently represented in the network, rabies is reported in both dogs and wildlife in neighbouring countries, with dogs the main reservoir of rabies to humans. Iraq's two largest neighbours, Turkey and Iran, both reported similar estimates of annual human rabies incidence of 0.02/million and 0.025/million respectively in 2009, and similar levels of PEP administration, at 1,700 and 2,290 per million population during the same year. Control measures in Turkey have effectively reduced dog rabies to restricted foci in urban areas. However, despite this reduction in dog rabies and implementation of control measures, rabies has re-emerged in the Aegean region, highlighting the complexities of controlling the disease [5].

There is minimal systematic surveillance for animal rabies in Iraq, and no laboratory confirmation of diagnosis. Vaccination is compulsory for dogs, but the majority of the urban dog population is considered ownerless, free-roaming and therefore presumed unvaccinated. There are no coordinated dog vaccination or sterilisation campaigns and dog population control has traditionally been attempted through culling, known to be an insufficient measure [12]. Rabies is also sporadically reported in wildlife, particularly in western regions, but the prevalence in wildlife, and the role of wildlife in maintenance and transmission of RABV to domestic animals and humans is poorly understood.

To address the lack of available data on rabies and to inform control strategies, sampling was initiated in the Baghdad region in April 2010. Here we report results of laboratory diagnosis and virus characterisation from these initial sampling efforts alongside official surveillance data for human rabies across Iraq.

## Methods

### Human rabies surveillance

Human rabies is notifiable in Iraq through regional public health offices in each of the 18 Governorates (provinces). Private and public health centres and hospitals report rabies cases based on a clinical definition of encephalitis, combined with hydrophobia and history of animal bite. There is no routine laboratory diagnosis undertaken. The regional public health offices report to the Zoonoses Section of the Centre for Disease Control (CDC) in Baghdad, who also collate post-exposure prophylaxis and reported animal bite numbers. These anonymized data on human rabies cases, animal bites and post-exposure prophylaxis were reviewed for the period 2001–2010. Analysis of the data was approved by the AHVLA Ethics Committee. Differences in rabies incidence between groups (age, sex and rural/urban habitation) were assessed using Chi-squared tests. Expected frequencies for age and area of habitation were taken from a separate recent household survey undertaken by others [13](Table 1).

**Table 1:** Reported rabies cases in Iraq 2001–2010 by age, sex and habitation.

Category	Population* (% of 30 million)	Rabies cases (% of 186 cases)	×^2^	*p* value
Age				
0–14	39.8	63		
15+	60.2	37		
			41.4	0.0001
Habitation				
Urban	70.9	17		
Rural	29.1	83		
			260	0.0001
Sex				
Male	50.2	89		
Female	49.8	11		
			113	0.0001

* population estimates from [13].

### Animal rabies sampling

#### Brain samples

Between April 2010 and July 2011, forty clinically ill animals (38 dogs, 2 cattle) in the Greater Baghdad area were euthanased by private veterinary surgeons or Iraqi State Veterinary Company staff. Cases were selected where rabies could not be ruled out on the basis of clinical signs. Reported signs included one or more of: abnormal behaviour or vocalisation, aggression, hyper-salivation and neurological signs. A pool of brain tissues including brain stem were removed at post mortem and stored frozen. All samples were transported frozen on dry-ice to the OIE Reference Laboratory at the Animal Health and Veterinary Laboratories Agency, Weybridge UK.

Brain samples were tested using a standard fluorescent antibody test (FAT) for lyssavirus antigen [14] and viral RNA was extracted from all brain samples in duplicate using enzymatic disruption (MELT, *Ambion*) according to the manufacturers protocol. RNA was reverse transcribed and a hemi-nested Reverse Transcriptase-Polymerase Chain Reaction (RT-PCR) was undertaken on each sample as described previously [15]. PCR products were purified (*Qiagen*) and sequenced as described previously [16]. At least one forward and one reverse primer were used to determine consensus sequences which were aligned using ClustalX2 (version 1.2).

Bayesian Markov Chain Monte Carlo (MCMC) phylogenetic analysis of the resulting consensus nucleoprotein sequences was implemented using the BEAST package (version 1.4.8) [17] with a panel of rabies viruses (RABVs) from neighbouring countries (Table S1). A relaxed molecular clock (uncorrelated lognormal) and GTR substitution model with a proportion of invariant sites were chosen over other evolutionary models based on comparison of multiple runs using Tracer (v1.4). A chain length of 10,000,000 was used and parameters were logged every 1,000 trees to ensure effective sample sizes were >200 and posterior distribution was normal. The maximum clade credibility (MCC) tree was chosen using TreeAnnotator (v 1.4.8) after the first 10% of trees were discarded and the resulting tree was visualised using FigTree (v1.2).

## Results

### Human rabies surveillance

**Figure 1:**
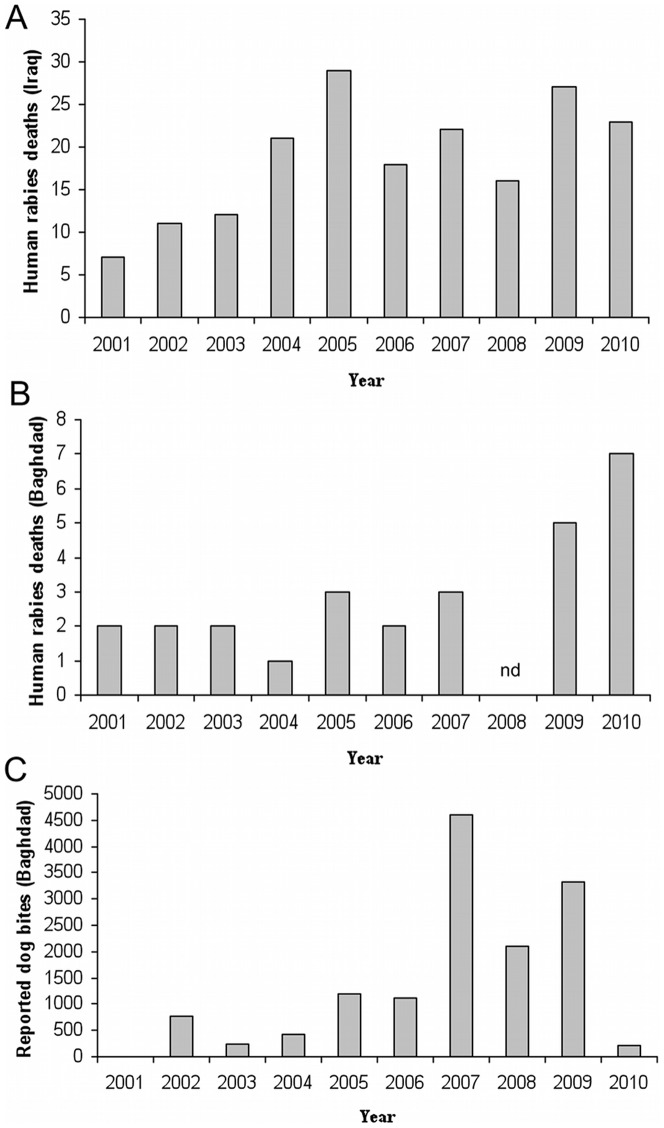
National statistics on human rabies incidence and dog bites. Data reported to National Zoonosis Centre, Baghdad from all 18 Governorate regional health offices. A. Annual reported incidence of human rabies deaths in Iraq, B Annual reported incidence of human rabies in Baghdad. C Annual reported dog bites in Baghdad. (nd = no data).

**Figure 2:**
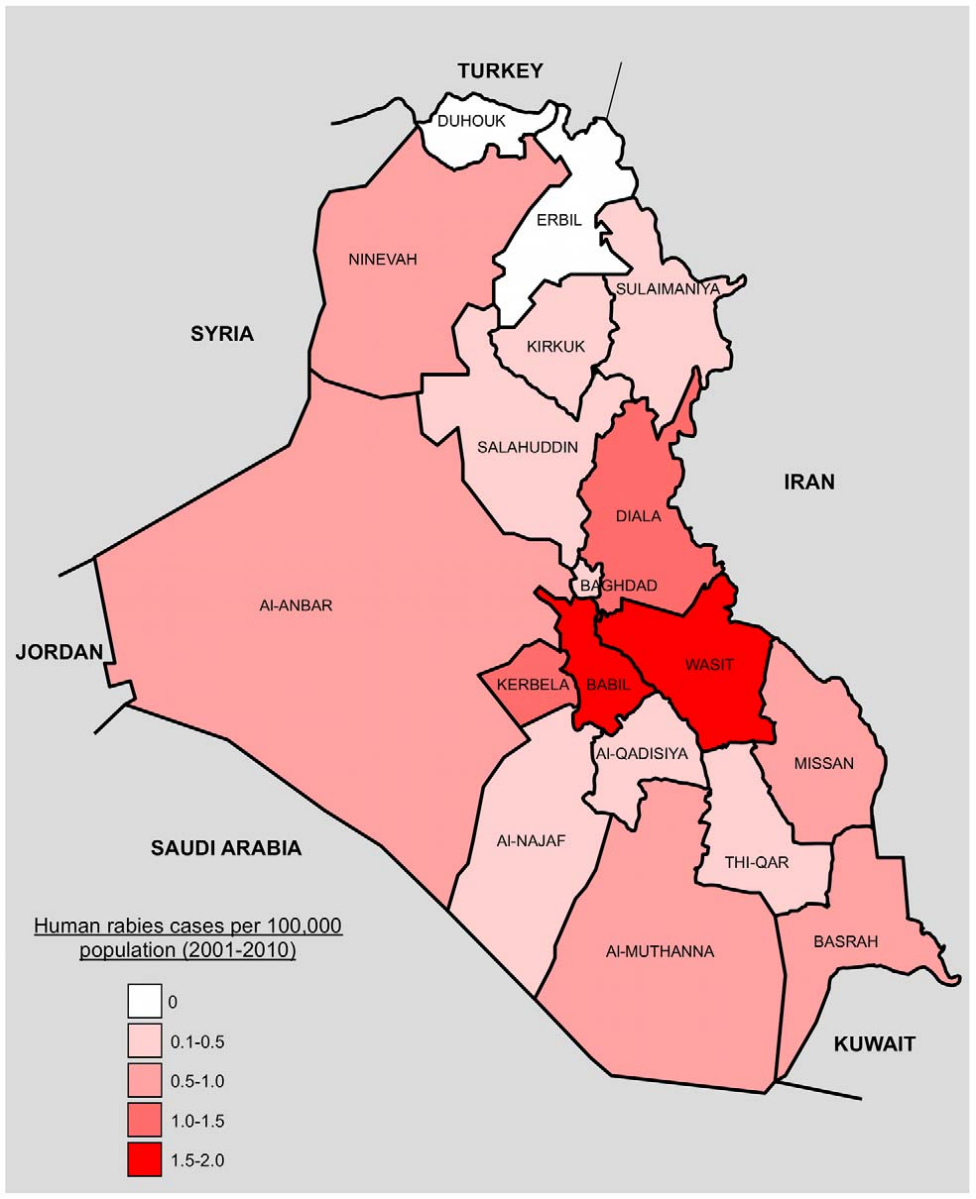
Map showing 18 Governorates of Iraq and bordering countries. Governorates are coloured by number of human rabies cases per 100,000 population 2001–2010. Population estimates were taken from a recent household survey [13]. Governorate and country boundaries are approximate.

Data on reported rabies cases were supplied by all 18 regional public health offices. In the 10 years between 2001 and 2010, there was an average of 17 (SD 6.9) human rabies cases reported annually in Iraq (Figure 1a). There was a three-fold increase in reported cases between 2003 and 2005 and, although the number of cases has varied from year to year, there has not been less than 15 cases reported per year since 2005. Human rabies incidence for Iraq during 2009 is estimated from these data at 0.89 deaths per million population, using a population estimate of 30 million [13] (Table 1). Children are over represented among rabies cases in Iraq. An estimated 40% of the population is under 15 years of age [13], yet 63% of cases occur in this age group (X^2^ = 48.4, p = 0.0001). Rabies is also more frequently reported in rural areas than urban areas, with 83% of cases reported in rural areas despite only 29% of the population living in rural areas [13] (X^2^ = 283, p = 0.0001). However, there has also been an apparent three-fold increase in the number of cases reported in Baghdad over the past ten years (albeit not statistically significant), with an average of 2 cases per year reported in between 2001 and 2002, and 6 cases reported per year between 2009 and 2010 (Figure 1b). There is an extreme bias towards males, with eight cases in males reported for every one case in a female despite a population sex ratio of 1∶1 [13] (X^2^ = 122, p = 0.0001). There is regional variation in the number of reported cases, with Governorates in the centre of the country reporting the highest incidence per 100,000 population during 2001–2010 (Figure 2 and Table S2).

Rabies prophylaxis is available in Iraq, although is not always initiated, and is rarely completed. The five-dose (Essen) regime is most frequently followed, and although a large proportion of dog bite victims receive the first vaccination (75%) a much lower number complete the full course (7%).

From 2002 to 2004 there were less than 1000 dog bites reported annually in Baghdad, corresponding to an incidence of 20 (95% CI 18.76–21.24) bites per 100,000 people, based on a population estimate of 5 million [18]. In the years between 2007 and 2010 the average had increased to 3300 bites reported per year (Figure 1c), corresponding to an annual incidence of 46 (95% CI 44.27–47.40) bites per 100,000 people, using a population estimate of 7.2 million [19] (paired incident rate test, p<0.0001) (Figure 1c).

### Animal rabies sampling

**Figure 3:**
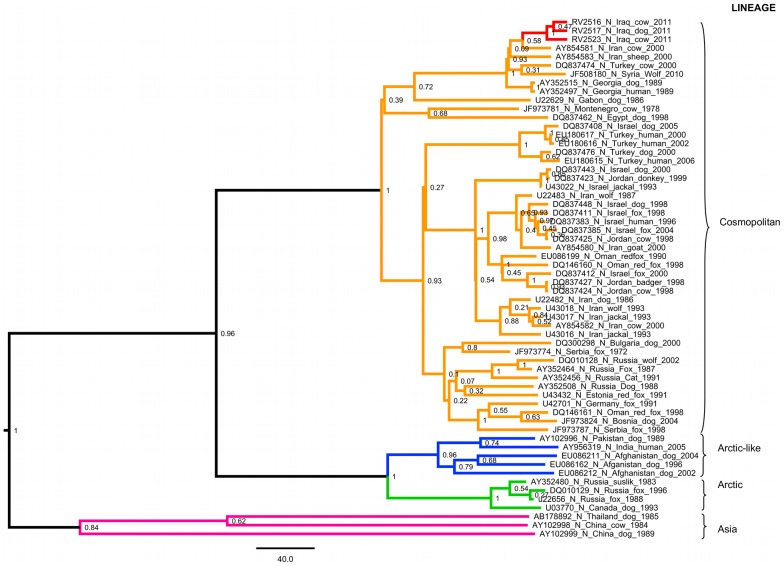
Bayesian phylogenetic analysis of 400 bp partial nucleoprotein sequences from Middle East. Analysis implemented in BEAST (v1.8), showing the relationship between viruses characterised in this study and published sequences from RABVs isolated in the Middle East (Table S1). Branches are coloured by lineage, with virus sequences from Baghdad in red. Scale bar represents 40 years.

Three out of 40 brain samples were positive for rabies virus by both FAT and RT-PCR. The three positive samples yielded unique partial nucleoprotein gene (N-gene) sequences (Genbank accession numbers JX524176-8). Phylogenetic analysis using a 400 base pair region of the N-gene showed that the viruses are closely related, forming a well supported clade separated from other published sequences (Figure 3). A relaxed molecular clock model applied to these data (assuming constant virus population size) suggests they share a common ancestor approximately 22 years ago (95% HPD 14–32 years) with viruses in the cosmopolitan lineage of RABV, from neighbouring countries including Turkey, Iran, and Syria.

## Discussion

Rabies is a preventable disease, and yet the data presented here demonstrate that it remains a significant public and animal health challenge in Iraq. All except two of the 18 Governorates reported human rabies cases during the period of study, indicating that rabies is endemic and widespread across the country. The reported incidence of human rabies far exceeds that reported by some neighbouring countries. Incidence during 2009 is estimated from these data at 0.89 deaths per million population, compared with 0.025 for Turkey and 0.02 for Iran [11]. There was an increase in reported cases for the whole country after 2003 and a three-fold increase in reported cases in Baghdad in the ten years between 2001 and 2010. This increase coincides with a period of intense conflict in Iraq, with the potential to have widespread direct and indirect consequences on disease control. These include well documented affects of the migration of health professionals and restrictions on travel, resources and sanitary conditions previously reported to cause increases in infectious diseases such as typhoid, measles and mumps in Iraq [6], [20]. As rabies is a zoonotic disease, with domestic dogs as primary reservoir host in many regions, changes to the free-roaming dog population will also have a large effect on human rabies incidence [3]. The effects of conflict on municipal services and disruption of human habitation are likely to have an impact on the urban dog population. The doubling of reported dog bites in Baghdad reported here coincide with the increase in human rabies cases and anecdotal reports of a mass expansion of the free-roaming dog population in Baghdad. It is likely therefore, that the increase in human rabies is due to an increase in the free roaming dog population and associated increase in dog bites. In addition to the increase in risk of zoonotic disease, the dog population increase has animal welfare implications. Historical approaches to manage dog populations in Baghdad have included pro-active culling in areas where large accumulations of free roaming dogs are reported. Although this temporarily results in fewer free roaming dogs, it is increasingly recognised that indiscriminate culling is not a long-term solution to dog population control, or to reducing rabies prevalence, has welfare implications and can make the situation worse [3], [12]. Animal birth control (ABC) programs, where sterilisation is combined with rabies vaccination, have proven effective in reducing or stabilising free-roaming dog populations, and also reducing rabies incidence [21], [22] but they may not be necessary in many socioeconomic settings [3], [23], [24], [25]. Therefore, where dog population management is used, it should be according to international guidelines and only after assessment of the local dog population [26]. Promotion of responsible dog ownership and appropriate legislative measures are also recommended as longer term solutions for controlling rabies in Iraq [12].

As with all studies using human rabies surveillance data, the data presented here have limitations. The low reported numbers of human rabies cases in Baghdad preclude robust statistical comparison, meaning that the apparent increase in cases in Baghdad could be the result of annual variation rather than a genuine increase in cases. In addition, human rabies cases are currently only diagnosed based on clinical data without laboratory confirmation. The incidence of other diseases with overlapping clinical presentations such as bacterial or viral encephalitis, could affect reporting if they are misdiagnosed as rabies, or if rabies cases are incorrectly diagnosed as other diseases [27], [28]. There are few published data on the incidence of encephalitidies in Iraq, but the region is at risk from pathogens, including tick borne encephalitis and West Nile virus [29], which could potentially be misdiagnosed as rabies. Finally, reporting effort may change overtime with changes in staff and resources, or between public health offices. Therefore we cannot control for possible change in reporting effort over time, or between Governorates.

The low numbers of bite victims completing a full course of post exposure prophylaxis is a significant risk to public health, but is a problem that is not unique to Iraq [30]. The reasons for failure to start or complete PEP given in similar contexts include failure to present for primary health care, cost of treatment, and misunderstanding of the risks [30]. Therefore increased public awareness and education may increase PEP uptake and reduce rabies deaths. To reduce the cost of PEP, a strategy adopted by some rabies endemic countries, is a reduced dose administered intradermally, which could be considered in Iraq [31]. The extreme male: female bias in cases is likely to be due to different occupational and domestic roles, causing different levels of exposure to potentially rabid animals but could also be affected by different tendency to present for treatment between the sexes [30], [32], [33]. Assuming negligible regional bias in reporting, the high numbers of cases in rural areas relative to the population means rural areas should be a priority for public awareness and rabies control.

There is no reported laboratory confirmation of rabies in Iraq, and circulating strains have not been previously characterised. In this study, brain samples for rabies diagnosis were analysed on an opportunistic basis from animals euthanased during the sampling period with one or more signs consistent with rabies. The prevalence of rabies in the sampled population (3/40) is lower than might be expected in a rabies endemic country. The clinical criteria for the sampled population included all animals with one or more sign consistent with rabies. Considering many signs of rabies are common to other diseases, this definition will include many non-rabid animals. All three positive samples were from Northern Baghdad, and two of the three positive cases were cattle. Cattle are considered dead-end hosts for rabies, and therefore these cases in cattle are likely to be spill over of the virus from undetected cases in dogs, or wildlife, in the area. This is supported by the phylogenetic evidence, suggesting these cases are from the same lineage circulating in dogs. The species bias towards large and economically important livestock animals provides supporting evidence to the hypothesis that the incidence is higher than these results suggest, and that many dog or wildlife cases go undetected or unreported. In addition, due to the necessary opportunistic nature of sampling, absence of cases in other areas does not imply freedom from rabies, and the same public and animal health interventions should be applied in all areas. Security concerns were a key restriction on sampling location and strategy in this study, and more comprehensive and systematic surveillance would be required to provide estimates of the incidence of animal rabies.

There are few isolates available from the region for phylogenetic analysis and hence sequence data from these cases therefore provide valuable information. A previously sequenced RABV reportedly from a dog in Iraq [34] was subsequently determined to have been incorrectly recorded, and it in fact originated in Afghanistan [Freuling, C., FLI Germany, pers. comm.]. Consequently, the present report is the first to characterise rabies strains in Iraq. The three sequences are unique over the region studied, but form a separate clade that diverged from related viruses approximately 22 (HPD 14–32) years ago. The most closely related viruses (with published sequence available) to this new Iraqi clade are from multiple different regions: Turkey, Iran, Syria and Georgia, which lie to the North East and West of Iraq. Turkey and Iran both have strains from other clades in the tree circulating contemporaneously with those in the Iraq clade. This information, combined with previously published evidence for a recent origin of RABV diversity in parts of the Middle East [10] suggests multiple introductions of rabies into the region, and likely transboundary movements of disease. Knowing the direction of any transboundary movements would help guide regional control policies, but would require a larger number of rabies sequences from the region to reduce sampling bias and increase resolution of phylogeographic analysis. These viruses circulating in the Middle East fall within the cosmopolitan lineage of RABV, suggested to have been spread worldwide by human movements in the 18^th^ and 19^th^ centuries [10], [35]–[37]. Estimates using these data for the most recent common ancestor for this European/Middle Eastern clade of the cosmopolitan lineage (101 years ago, HPD 59–114) concur with previous estimates [10].

These close relationships between viruses from Iraq and neighbouring countries reiterate that rabies does not respect cultural or political barriers and elimination of rabies must be approached at a regional level, with global cooperation from international veterinary (OIE/FAO) and health (WHO) providers. This is already being addressed through NGOs, and the MEEREB network but will require consistent commitment and resources [11], [38]. Despite the on-going conflict, notable efforts, with international support, are being made to improve primary health care in addition to hospital based healthcare in Iraq [39], [40]. In addition, collaboration between environmental, human and veterinary health departments is essential at a local level to eliminate rabies in dogs, and thereby reduce the risk to humans [41].

## Supporting Information

Table S1 **Country, species of origin, and year of isolation for sixty rabies virus nucleoprotein sequences used for phylogenetic analysis.**
(DOC)

Table S2 **Human rabies cases reported by all 18 Governorate public health offices 2001–2010.**
(DOC)

## References

[1] Livingstone A (1988) The Isin Dog House Revisited. Journal of cuneiform Studies 40: 54–60.

[2] Theodorides J (1986) Histoire de la Rage. In: Masson, editor. Cave Canem. Paris. pp.289.

[3] Zinsstag J, Durr S, Penny MA, Mindekem R, Roth F, et al (2009) Transmission dynamics and economics of rabies control in dogs and humans in an African city. Proceedings of the National Academy of Sciences of the United States of America 106: 14996–15001. 19706492 10.1073/pnas.0904740106 PMC2728111

[4] Davies MC, Englert ME, Sharpless GR, Cabasso VJ (1963) The Electron Microscopy of Rabies Virus in Cultures of Chicken Embryo Tissues. Virology 21: 642–651. 14100615 10.1016/0042-6822(63)90238-1

[5] Johnson N, Un H, Fooks AR, Freuling C, Muller T, et al (2010) Rabies epidemiology and control in Turkey: past and present. Epidemiol Infect 138: 305–312. 19814851 10.1017/S0950268809990963

[6] Wilson JF (2004) The health care revival in Iraq. Annals of internal medicine 141: 825–828. 15545690 10.7326/0003-4819-141-10-200411160-00027

[7] Amin NM, Khoshnaw MQ (2003) Medical education and training in Iraq. Lancet 362: 1326. 14579817 10.1016/s0140-6736(03)14580-1

[8] Seimenis A (2008) The rabies situation in the Middle East. Developments in biologicals 131: 43–53. 18634465

[9] David D, Yakobson B, Smith JS, Stram Y (2000) Molecular epidemiology of rabies virus isolates from Israel and other middle- and Near-Eastern countries. J Clin Microbiol 38: 755–762. 10655381 10.1128/jcm.38.2.755-762.2000 PMC86197

[10] David D, Hughes GJ, Yakobson BA, Davidson I, Un H, et al (2007) Identification of novel canine rabies virus clades in the Middle East and North Africa. J Gen Virol 88: 967–980. 17325371 10.1099/vir.0.82352-0

[11] Aylan O, El-Sayed AF, Farahtaj F, Janani AR, Lugach O, et al (2011) Report of the first meeting of the middle East and eastern europe rabies expert bureau, istanbul, Turkey (june 8–9, 2010). Advances in preventive medicine 2011: 812515. 21991443 10.4061/2011/812515 PMC3173715

[12] Lembo T (2012) The blueprint for rabies prevention and control: a novel operational toolkit for rabies elimination. PLoS neglected tropical diseases 6: e1388. 22389727 10.1371/journal.pntd.0001388 PMC3289591

[13] World Bank (2008) *Data tables* In: Iraq household socio-economic survey IHSES - 2007 : tabulation report. Washington D.C.: The Worldbank. Available: http://documents.worldbank.org/curated/en/2008/12/10192867/iraq-household-socio-economic-survey-ihses-2007-tabulation-report-vol-2-3-data-tables *Accessed: 25 July 2012.*

[14] World Organisation for Animal Health [OIE] (2011) Rabies. Manual of Diagnostic Tests and Vaccines for Terrestrial Animals, Chapter 2.1.13. Paris: World Organisation for Animal Health. Available: http://www.oie.int/fileadmin/Home/eng/Health_standards/tahm/2.01.13_RABIES.pdf *Accessed: 25 July 2012.*

[15] Marston DA, McElhinney LM, Johnson N, Muller T, Conzelmann KK, et al (2007) Comparative analysis of the full genome sequence of European bat lyssavirus type 1 and type 2 with other lyssaviruses and evidence for a conserved transcription termination and polyadenylation motif in the G-L 3′ non-translated region. J Gen Virol 88: 1302–1314. 17374776 10.1099/vir.0.82692-0

[16] Johnson N, McElhinney LM, Smith J, Lowings P, Fooks AR (2002) Phylogenetic comparison of the genus Lyssavirus using distal coding sequences of the glycoprotein and nucleoprotein genes. Arch Virol 147: 2111–2123. 12417947 10.1007/s00705-002-0877-4

[17] Drummond AJ, Ho SY, Phillips MJ, Rambaut A (2006) Relaxed phylogenetics and dating with confidence. PLoS Biol 4: e88. 16683862 10.1371/journal.pbio.0040088 PMC1395354

[18] Roberts L, Lafta R, Garfield R, Khudhairi J, Burnham G (2004) Mortality before and after the 2003 invasion of Iraq: cluster sample survey. Lancet 364: 1857–1864. 15555665 10.1016/S0140-6736(04)17441-2

[19] Burnham G, Hoe C, Hung YW, Ferati A, Dyer A, et al (2011) Perceptions and utilization of primary health care services in Iraq: findings from a national household survey. BMC international health and human rights 11: 15. 22176866 10.1186/1472-698X-11-15 PMC3266634

[20] Dyer O (2004) Infectious diseases increase in Iraq as public health service deteriorates. BMJ 329: 940. 10.1136/bmj.329.7472.940-a PMC524138 15499110

[21] Reece JF, Chawla SK (2006) Control of rabies in Jaipur, India, by the sterilisation and vaccination of neighbourhood dogs. Vet Rec 159: 379–383. 16980523 10.1136/vr.159.12.379

[22] Totton SC, Wandeler AI, Zinsstag J, Bauch CT, Ribble CS, et al (2010) Stray dog population demographics in Jodhpur, India following a population control/rabies vaccination program. Preventive veterinary medicine 97: 51–57. 20696487 10.1016/j.prevetmed.2010.07.009

[23] Coleman PG, Dye C (1996) Immunization coverage required to prevent outbreaks of dog rabies. Vaccine 14: 185–186. 8920697 10.1016/0264-410x(95)00197-9

[24] Lembo T, Hampson K, Kaare MT, Ernest E, Knobel D, et al (2010) The feasibility of canine rabies elimination in Africa: dispelling doubts with data. PLoS Negl Trop Dis 4: e626. 20186330 10.1371/journal.pntd.0000626 PMC2826407

[25] Morters MK, Restif O, Hampson K, Cleaveland S, Wood JL, et al (2012) Evidence-based control of canine rabies: a critical review of population density reduction. The Journal of animal ecology 82: 6–14. 23004351 10.1111/j.1365-2656.2012.02033.x PMC3579231

[26] World Organisation for Animal Health [OIE] (2011) Stray Dog Population Control. In: Terrestrial Animal Health Code. Paris: World Organisation for Animal Health. Available: http://www.oie.int/doc/ged/D9926.PDF *Accessed: 25 July 2012*.

[27] Whitley RJ, Gnann JW (2002) Viral encephalitis: familiar infections and emerging pathogens. Lancet 359: 507–513. 11853816 10.1016/S0140-6736(02)07681-X

[28] Mallewa M, Fooks AR, Banda D, Chikungwa P, Mankhambo L, et al (2007) Rabies encephalitis in malaria-endemic area, Malawi, Africa. Emerg Infect Dis 13: 136–139. 17370529 10.3201/eid1301.060810 PMC2725806

[29] Solomon T, Cardosa MJ (2000) Emerging arboviral encephalitis. Newsworthy in the West but much more common in the East. BMJ 321: 1484–1485. 11118162 10.1136/bmj.321.7275.1484 PMC1119205

[30] Both L, Banyard AC, van Dolleweerd C, Horton DL, Ma JK, et al (2012) Passive immunity in the prevention of rabies. The Lancet infectious diseases 12: 397–407. 22541629 10.1016/S1473-3099(11)70340-1

[31] Verma R, Khanna P, Prinja S, Rajput M (2011) Intra-dermal administration of rabies vaccines in developing countries: at an affordable cost. Human vaccines 7: 792–794. 21734465 10.4161/hv.7.7.15410

[32] Singh J, Jain DC, Bhatia R, Ichhpujani RL, Harit AK, et al (2001) Epidemiological characteristics of rabies in Delhi and surrounding areas, 1998. Indian pediatrics 38: 1354–1360. 11752732

[33] Tenzin Dhand NK, Ward MP (2012) Anthropogenic and environmental risk factors for rabies occurrence in Bhutan. Preventive veterinary medicine 107: 21–26. 22673581 10.1016/j.prevetmed.2012.05.003

[34] Kuzmin IV, Hughes GJ, Botvinkin AD, Gribencha SG, Rupprecht CE (2008) Arctic and Arctic-like rabies viruses: distribution, phylogeny and evolutionary history. Epidemiol Infect 136: 509–519. 17599781 10.1017/S095026880700903X PMC2870842

[35] Smith JS, Orciari LA, Yager PA, Seidel HD, Warner CK (1992) Epidemiologic and historical relationships among 87 rabies virus isolates as determined by limited sequence analysis. J Infect Dis 166: 296–307. 1634801 10.1093/infdis/166.2.296

[36] Badrane H, Tordo N (2001) Host switching in Lyssavirus history from the Chiroptera to the Carnivora orders. J Virol 75: 8096–8104. 11483755 10.1128/JVI.75.17.8096-8104.2001 PMC115054

[37] Bourhy H, Reynes JM, Dunham EJ, Dacheux L, Larrous F, et al (2008) The origin and phylogeography of dog rabies virus. J Gen Virol 89: 2673–2681. 18931062 10.1099/vir.0.2008/003913-0 PMC3326349

[38] World Health Organisation [WHO]/World Organisation for Animal Health [OIE] (2008) Inter-country expert workshop on protecting humans form domestic and wildlife rabies in the Middle East. Amman, Jordan. Workshop report. Available: http://www.rr-middleeast.oie.int/download/pdf/ammanrabies2.pdf, *Accessed 20^th^ July 2012*.

[39] University Research Co., LLC (URC), (2011). URC Awarded $74.8 Million US Agency for International Development Contract to Support Iraqi Ministry of Health Achieve Quality Primary Health Care (press release March 2011). Available: http://www.urc-chs.com/resource?ResourceID=380 *Accessed: July 2012.*

[40] United Nations Food and Agriculture Organisation [FAO] (2008) Iraq Veterinary Services and Zoonotic Diseases Projects. Project: A5 - 19, United Nations Development Group Iraq Trust Fund, Project Summary Sheet, updated June 2008. Available: http://www.faoiraq.org/images/word/Zoonatic.pdf *Accessed: July 20 2012*.

[41] Zinsstag J, Schelling E, Roth F, Bonfoh B, de Savigny D, et al (2007) Human benefits of animal interventions for zoonosis control. Emerging infectious diseases 13: 527–531. 17553265 10.3201/eid1304.060381 PMC2725951

