# Supplementary material for: Rabies in Iraq: Trends in Human Cases 2001–2010 and Characterisation of Animal Rabies Strains from Baghdad
Source: PLoS Negl Trop Dis. 2013 Feb 28;7(2):e2075. doi: 10.1371/journal.pntd.0002075 (PMC3585036; doi:10.1371/journal.pntd.0002075)
Supplement: Table S1 — Country, species of origin, and year of isolation for sixty rabies virus nucleoprotein sequences used for phylogenetic analysis. (DOC) [file pntd.0002075.s001.doc]

**Table S1.** Country, species of origin, and year of isolation for sixty rabies virus nucleoprotein sequences used for phylogenetic analysis.

| **Accession number** | **Country** | **Species** | **Year** |
| --- | --- | --- | --- |
| JX524176* | Iraq | cow | 2011 |
| JX524177* | Iraq | dog | 2011 |
| JX524178* | Iraq | cow | 2011 |
| JF508180 | Syria | wolf | 2010 |
| EU180615 | Turkey | human | 2006 |
| AY956319 | India | human | 2005 |
| DQ837408 | Israel | dog | 2005 |
| EU086211 | Afghanistan | dog | 2004 |
| DQ837385 | Israel | fox | 2004 |
| JF973824 | Bosnia | dog | 2004 |
| EU086212 | Afghanistan | dog | 2002 |
| DQ010128 | Russia | wolf | 2002 |
| EU180616 | Turkey | human | 2002 |
| AY854581 | Iran | cow | 2000 |
| AY854583 | Iran | sheep | 2000 |
| DQ837474 | Turkey | cow | 2000 |
| DQ837412 | Israel | fox | 2000 |
| AY854580 | Iran | goat | 2000 |
| AY854582 | Iran | cow | 2000 |
| DQ837443 | Israel | dog | 2000 |
| EU180617 | Turkey | human | 2000 |
| DQ837476 | Turkey | dog | 2000 |
| DQ300298 | Bulgaria | dog | 2000 |
| DQ837423 | Jordan | donkey | 1999 |
| DQ837425 | Jordan | cow | 1998 |
| DQ837411 | Israel | fox | 1998 |
| DQ837448 | Israel | dog | 1998 |
| DQ837424 | Jordan | cow | 1998 |
| DQ837427 | Jordan | badger | 1998 |
| DQ146160 | Oman | fox | 1998 |
| DQ146161 | Oman | fox | 1998 |
| JF973787 | Serbia | fox | 1998 |
| DQ837462 | Egypt | dog | 1998 |
| DQ010129 | Russia | fox | 1996 |
| EU086162 | Afghanistan | dog | 1996 |
| DQ837383 | Israel | human | 1996 |
| U03770 | Canada | dog | 1993 |
| U43017 | Iran | jackal | 1993 |
| U43018 | Iran | wolf | 1993 |
| U43016 | Iran | jackal | 1993 |
| U43022 | Israel | jackal | 1993 |
| AY352456 | Russia | cat | 1991 |
| U43432 | Estonia | fox | 1991 |
| U42701 | Germany | fox | 1991 |
| EU086199 | Oman | fox | 1990 |
| AY102996 | Pakistan | dog | 1989 |
| AY352497 | Georgia | human | 1989 |
| AY352515 | Georgia | dog | 1989 |
| AY102999 | China | dog | 1989 |
| u22656 | Russia | fox | 1988 |
| AY352508 | Russia | dog | 1988 |
| U22483 | Iran | wolf | 1987 |
| AY352464 | Russia | fox | 1987 |
| U22629 | Gabon | dog | 1986 |
| U22482 | Iran | dog | 1986 |
| AB178892 | Thailand | dog | 1985 |
| AY102998 | China | cow | 1984 |
| AY352480 | Russia | ground squirrel | 1983 |
| JF973781 | Montenegro | cow | 1978 |
| JF973774 | Serbia | fox | 1972 |

* sequences derived in this study
