# Supplementary material for: Rabies in Iraq: Trends in Human Cases 2001–2010 and Characterisation of Animal Rabies Strains from Baghdad
Source: PLoS Negl Trop Dis. 2013 Feb 28;7(2):e2075. doi: 10.1371/journal.pntd.0002075 (PMC3585036; doi:10.1371/journal.pntd.0002075)
Supplement: Table S2 — Human rabies cases reported by all 18 Governorate public health offices 2001–2010. (DOC) [file pntd.0002075.s002.doc]

Table S2. Human rabies cases reported by all 18 Governorate public health offices 2001-2010

| **Governorate** | **Total cases 2001-2010** | **Population*** | **Cases/100,000** |
| --- | --- | --- | --- |
| DUHOUK | 0 | 895000 | 0.00 |
| ERBIL | 0 | 1409000 | 0.00 |
| SULAIMANIYA | 4 | 1574000 | 0.25 |
| THI-QAR | 6 | 1687000 | 0.36 |
| BAGHDAD | 27 | 6995000 | 0.39 |
| SALAHUDDIN | 5 | 1158000 | 0.43 |
| KIRKUK | 5 | 1129000 | 0.44 |
| Al-NAJAF | 5 | 1113000 | 0.45 |
| Al-QADISIYA | 5 | 1033000 | 0.48 |
| NINEVAH | 16 | 2820000 | 0.57 |
| Al-ANBAR | 9 | 1427000 | 0.63 |
| BASRAH | 17 | 2408000 | 0.71 |
| Al-MUTHANNA | 5 | 650000 | 0.77 |
| MISSAN | 9 | 944000 | 0.95 |
| KERBELA | 10 | 902000 | 1.11 |
| DIALA | 15 | 1323000 | 1.13 |
| WASIT | 17 | 1056000 | 1.61 |
| BABIL | 31 | 1574000 | 1.97 |

* population estimates taken from (13).
